# Supplementary material for: Methylenetetrahydrofolate Reductase Polymorphism (rs1801133) and the Risk of Hypertension among African Populations: A Narrative Synthesis of Literature
Source: Genes (Basel). 2022 Apr 1;13(4):631. doi: 10.3390/genes13040631 (PMC9027465; doi:10.3390/genes13040631)
Supplement: Supplementary file 1 [file genes-13-00631-s001.zip › Search Terms MTHFR 24-01-22.pdf]

Supplementary File S1

SEARCH TERMS

(((methylenetetrahydrofolate reductase gene) OR (rs1801133)) OR (MTHFR) AND ((("high blood pressure"[Title/Abstract] AND 1984/01/01:2121/12/31[Date-Publication]) OR ("hypertens*"[Title/Abstract] AND 1984/01/01:2121/12/31[Date-Publication]) OR ("uncontrolled hypertension"[Title/Abstract] AND 1984/01/01:2121/12/31[Date-Publication]) OR ("resistant hypertension"[Title/Abstract] AND 1984/01/01:2121/12/31[Date-Publication])) AND 1984/01/01:2021/12/31[Date-Publication]) AND 1984/01/01:2021/12/31[Date-Publication] AND (("pharmacogenomic*"[Title/Abstract] OR "mutat*"[Title/Abstract] OR "snp"[Title/Abstract] OR "single nucleotide polymorphism"[Title/Abstract] OR "sequence variants"[Title/Abstract] OR "variation*"[Title/Abstract] OR "varian*"[Title/Abstract] OR "genetic marker*"[Title/Abstract] OR "polymorph*"[Title/Abstract] OR "genes"[MeSH Terms] OR "genes"[All Fields] OR "gene"[All Fields]) AND 1984/01/01:2021/12/31[Date-Publication] AND 1984/01/01:2021/12/31[Date - Publication])) AND (1984/1/1:2021/12/31[pdat])
